# Estimation of gut microbiome motif associated with active tuberculosis - A case control study

**DOI:** 10.6026/973206300222083

**Published:** 2026-04-30

**Authors:** Meenal Jain, Tanya Jain, Rajat Nayak, Talha Saad, Akash Wamanrao Karale, Mohammad Ilyas, Ravikant Arjariya

**Affiliations:** 1 Department of Anaesthesiology, Bundelkhand Government Medical College Sagar, Madhya Pradesh, India; 2 Department of Anaesthesiology, R.D. Gardi Medical College, Ujjain, Madhya Pradesh, India; 3 Department of Emergency Medicine, Bundelkhand Government Medical College Sagar, Madhya Pradesh, India; 4 Department of Respiratory Medicine, Bundelkhand Medical College Sagar, Madhya Pradesh, India; 5 Department of surgery, Bundelkhand Medical College, Sagar, Madhya Pradesh, India; 6 Department of Anaesthesia, Bundelkhand medical College Sagar, Madhya Pradesh, India; 7 Department of Physiology, Bundelkhand Government Medical College, Sagar, Madhya Pradesh, India

**Keywords:** Tuberculosis (TB), gut microbiota, gut-lung axis, dysbiosis

## Abstract

Tuberculosis (TB) is the most tenacious health issue affecting individuals globally. Therefore, it is of interest to estimate gut microbiota motifs associated with the outbreak of active TB by incorporating the insights from human case-control data. 106 respondents were categorised into 2 groups: group A with TB and group B controls with 53 respondents in each category. The Shannon diversity index was substantially diminished in affected individuals. The active TB cases exhibited substantially decreased micro-biomes (P < 0.001). Thus, we show the need to develop microbiome-targeted strategies to reduce the incidence of active TB cases and improve their effective management.

## Background:

The infectious disease that causes the greatest death rates worldwide is the contraction of Mycobacterium tuberculosis bacteria, turning into active tuberculosis (TB) infection. It has been disclosed that more than 10 million cases and 1 million deaths occur per year despite its control measures worldwide. One-third of the individuals globally contract with Mycobacterium tuberculosis, but only a few progresses into active TB disease [1]. This disparity reveals that immunity plays a major role in disease progression. The conventional elements like malnutrition, diabetes, smoking status, HIV infection and poor socio-economic status are the well-known risk factors for the causation of TB. These risk factors are not capable of clarifying why a few individuals progressed to active TB. It has been disclosed that gut microbiota carries a modifiable risk factor for immunosuppression in individuals, resulting in progression into active TB disease [2]. The Immune function, its metabolic regulation and inflammatory responses are impacted by the micro-biomes residing in an individual's gastrointestinal tract region. There are alterations in the immune defence response in the lung region through the gastrointestinal-lung axis due to the microbiota metabolism and the circulation of immune mediators [3, 4]. Dysbiosis, defined as a modification in the gut microbiota, has been identified as a factor in asthma, chronic obstructive pulmonary disease and viral pneumonias. Various studies have reported consistent observations on the progression of TB [5, 6]. The understanding of gut microbiota motifs is linked to progression to active TB, supporting a biomarker for early diagnosis and generating opportunities for microbiota-targeted treatment. Therefore, it is of interest to investigate the motifs of gastrointestinal micro-biomes associated with the progression of active TB infection.

## Materials and Methods:

## Study design and sample:

Case-control research was conducted on 106 subjects over 18 months, from January 2023 to June 2024. The 53 active TB cases were enrolled in the current investigation and 53 unaffected respondents were enrolled. The respondents ranging between the ages of 18 and 60 years old with lab-proven infectious disorder of the lung, sputum smear, culture or Gene-Xpert positive; exhibiting signs and symptoms of coughing for weeks, fever, weight loss and patches or holes in chest x-ray (CXR); along with a history of taking any medicines were the inclusions for the present case-control research. The respondents with no signs and symptoms of TB infection, HIV positive cases, carcinoma cases taking immunosuppression medicines and having a history of any antibiotics or steroids in the last month were excluded from the present research.

## Ethical considerations:

Informed consent was obtained from all respondents. The respondents' data were kept private and secure.

## Sample size estimation:

The sample size was estimated by implementing the equation as follows (see PDF):

On substituting, 1.96 for our 5% alpha (two-tailed), 0.84 for 80% power and the p1/p2 guesses yielding 53 respondents in each group.

## Data collection:

Data were collected through thorough history-taking of the respondents. The age, status of smoking and alcohol use, diabetic and hypertensive status of the respondents, BCG vaccination status, TB timelines, scoring of sputum culture and CXR were assessed. The intake of medicines that affect gut fauna, such as antacids, analgesics and anti-diabetic drugs, was noted in the questionnaire. Fresh stool samples were collected from all respondents using sterile, labelled containers under standardized conditions. The specimens were readily sent to the testing facility while maintained at -80°C, awaiting analysis to retain bacterial viability. Pursuant to the company's directions, bacterial genomic DNA was extracted from the specimen using the stool DNA extraction kit. The NanoDrop spectrophotometer was then used to analyse DNA sterility and concentration. By integrating the distinctive primers, the V3-V4 hypervariable segments of the 16S rRNA gene were boosted and harmonized on an Illumina MiSeq platform. The unprocessed sequencing reads underwent comprehensive analysis to remove low-quality sequences, were edited to meet requirements and were checked for chimeric reads. Sequence data were processed using QIIME2 software. Following analysis, genomes were organized into functional taxonomic entities and the SILVA database served as the basis for taxonomic categorization. The Shannon diversity index was used to assess alpha diversity in bacteria and Bray-Curtis dissimilarity indices were used to assess beta diversity. At the phylum level, the relative numbers of different bacterial species were investigated.

## Statistical analysis:

Demographic variables were analysed using appropriate descriptive statistics. Depending on the layout of the collected information, Mann-Whitney U test was used to compare continuous variables. The Mann-Whitney U test was used to assess differences in microbial indices and abundance relationships across groups. Differentially abundant taxa have been discovered incorporating LEfSe (Linear discriminant analysis Effect Size).

## Results:

106 participants (53 active TB cases, 53 controls) were analysed with no dropouts. Groups matched well on age (TB: 34.2±10.1 years; controls: 35.1±11.2; P=0.72) and sex (45% vs. 47% female; P=0.84), but TB cases showed lower BMI (19.2±2.8 vs. 23.1 ±3.4 kg/m^2^; P<0.001) and more smokers (28% vs. 13%; P=0.04), as expected in disease. Gut microbial alpha diversity, assessed using the Shannon diversity index and was significantly lower in individuals with active tuberculosis compared to unaffected respondents (Table 1 - see PDF). Group respondents exhibited substantially diminished micro-biomes, highlighting pronounced gut dysbiosis (P < 0.001). There was a notable disparity among groups in microbial phylum-level composition. The respondents in group A exhibited heightened levels of Proteobacteria and Actinobacteria. Firmicutes and Bacteroides were augmented in group B. Conversely, the reduced abundance of Firmicutes and Bacteroidetes - taxa commonly associated with gut homeostasis and immune modulation-was a consistent finding among TB cases. Numerous microbial species have been identified as exceedingly abundant in both groups by LEfSe analysis. Those suffering from TB have a greater tendency to possess species belonging to the Proteobacteria, whilst healthy responders are more prone to have commensal bacteria associated with immune response and metabolic regulation.

## Discussion:

The current research investigation illuminates subtle variations in the composition of the intestinal microbiome among responders with ongoing pulmonary TB and those without. The findings highlight significant differences in microbial diversity and the relative abundance of major bacterial phyla, reinforcing the growing concept of the gut-lung axis and its relevance in tuberculosis pathogenesis. One of the most prominent observations in this study was the significantly reduced alpha diversity among tuberculosis patients. Similar reductions in gut microbial diversity among affected individuals have been reported by Hu *et al.* who demonstrated distinct microbiome signatures that differentiate pulmonary TB patients from healthy individuals [7]. Compared with healthy individuals, Wang *et al.* reported reduced bacterial diversity in severe cases, highlighting the relationship between dysbiosis and disease progression [8]. The gastrointestinal micro-biomes can affect host gene expression via epigenetic mechanisms, as uncovered in various studies [9, 10, 11-12]. In contrast, numerous studies have shown only minor or marginal variation in alpha diversity, especially among individuals predisposed to different dietary or ecological factors. In this regard, Cao *et al.* found that changes in bacterial biodiversity were more pronounced following the administration of anti- tuberculosis medications than at the outset, suggesting that the drugs themselves can be an important component in the development of dysbiosis [13]. This clarifies that disparities exist in the research design, the research region, the sequencing depth and the host lifestyle. Nevertheless, the consistent observation of reduced diversity in the present study supports the hypothesis that gut microbial imbalance is a characteristic feature of active TB.

The affected individuals exhibited minimally abundant Firmicutes in juxtaposition to the unaffected group. Reduced Firmicutes abundance has previously been reported by Zheng *et al.* who linked depletion of beneficial gut bacteria to impaired immune tolerance and heightened inflammatory states [14]. Conversely, Segal and Blaser reported relatively stable Firmicutes levels in certain respiratory disease cohorts, suggesting that not all inflammatory lung conditions exhibit uniform changes in the gut microbiome [15]. This information might indicate that reductions in Firmicutes might be more substantial in infections with systemic immunological manifestations, such as TB, than in confined lung conditions. More importantly, the present investigation demonstrated that individuals with TB had a low proportion of Bacteroidetes. A decreased Bacteroidetes concentration was shown by Schirmer *et al.* to be correlated with diminished cytokine generation, which may jeopardize hosts' ability to defend against intracellular pathogens such as Mycobacterium TB [16]. Dubourg *et al.* exhibited diminished disparities in Bacteroidetes abundance between the two groups [17]. These contrasting findings suggest that methodological variability and population heterogeneity may influence observed microbial patterns. Proteobacteria were substantially elevated in the active disease group compared with unaffected individuals in the present study. Proteobacteria are often considered a microbial signature of dysbiosis and are enriched in pro-inflammatory states. Researchers described Proteobacteria expansion as a marker of microbial imbalance associated with epithelial dysfunction and immune activation [18]. Wipperman *et al.* additionally identified higher amounts of this phylum in TB sufferers, particularly after being exposed to drugs [19]. On the contrary, some evidence suggests that Proteobacteria concentration may not be a reliable indicator of illness; instead, it may be transient or affected by acute inflammatory conditions. Proteobacteria concentration varied across multiple phases of medical treatment, as demonstrated by Cao *et al.* highlighting that host immune system status and generalized inflammation, rather than TB alone, might have been the reason for these fluctuations [13]. The current investigation highlighted that Actinobacteria were substantially more prevalent in TB cases, which was similar to the study of Sahu *et al.* [20]. This phylum comprises both harmful and beneficial taxa and the role of its members in tuberculosis remains unresolved. Parallel spikes in Actinobacteria were observed by Hu *et al.* [7] in people with TB, signalling an immune-enhancing or compensatory effect. Conversely, Wang *et al.* [8] observed reduced Actinobacteria levels in certain TB cohorts, highlighting inconsistencies across studies. This disparate information suggests that Actinobacteria shifts could be contingent on immune response, dietary status and environmental exposure, rather than constituting a homogeneous, distinctive characteristic of TB. The notion that gastrointestinal dysbiosis could contribute to susceptibility to active TB or symbolize global immunological dysfunction attributable to the illness is reinforced by the modified intestinal microbiome profile discovered in the present investigation. To determine causality and assess whether microbiome-focused therapies, particularly probiotics or dietary modifications, could be incorporated as auxiliary approaches for the prevention and treatment of TB, long-term analyses are needed.

## Conclusion:

Bacteria-targeted methods might serve as auxiliary approaches in the treatment of TB and gastrointestinal microbial characteristics might serve as potential indicators of illness susceptibility or progression. Thus, data helps in linking gastrointestinal microbes to immune system reactions in infectious illnesses. Further, the vital role of gastrointestinal microbiota irregularities in active pulmonary TB is shown.
